# Preloading Effect on Strengthening Efficiency of RC Beams Strengthened with Non- and Pretensioned NSM Strips

**DOI:** 10.3390/polym10020145

**Published:** 2018-02-03

**Authors:** Renata Kotynia, Marta Przygocka

**Affiliations:** Department of Concrete Structures, Lodz University of Technology, 90-924 Lodz, Poland; marta.przygocka@wp.pl

**Keywords:** flexural strengthening, NSM, CFRP, pretensioning, preloading, load bearing capacity, serviceability limit state

## Abstract

The near surface mounted (NSM) technique has been shown to be one of the most promising methods for upgrading reinforced concrete (RC) structures. Many tests carried out on RC members strengthened in flexure with NSM fiber-reinforced polymer (FRP) systems have demonstrated greater strengthening efficiency than the use of externally-bonded (EB) FRP laminates. Strengthening with simultaneous pretensioning of the FRP results in improvements in the serviceability limit state (SLS) conditions, including the increased cracking moment and decreased deflections. The objective of the reported experimental program, which consisted of two series of RC beams strengthened in flexure with NSM CFRP strips, was to investigate the influence of a number of parameters on the strengthening efficiency. The test program focused on an analysis of the effects of preloading on the strengthening efficiency which has been investigated very rarely despite being one of the most important parameters to be taken into account in strengthening design. Two preloading levels were considered: the beam self-weight only, which corresponded to stresses on the internal longitudinal reinforcement of 25% and 14% of the yield stress (depending on a steel reinforcement ratio), and the self-weight with the additional superimposed load, corresponding to 60% of the yield strength of the unstrengthened beam and a deflection equal to the allowable deflection at the SLS. The influence of the longitudinal steel reinforcement ratio was also considered in this study. To reflect the variability seen in existing structures, test specimens were varied by using different steel bar diameters. Finally, the impact of the composite reinforcement ratio and the number of pretensioned FRP strips was considered. Specimens were divided into two series based on their strengthening configuration: series “A” were strengthened with one pretensioned and two non-pretensioned carbon FRP (CFRP) strips, while series “B” were strengthened with two pretensioned strips. Experimental tests illustrated promising results at ultimate and serviceability limit state conditions. A significant gain of the load bearing capacity, in the range between 56% and 135% compared to the unstrengthened beams, was obtained. Tensile rupture of the NSM CFRP strips was achieved, confirming full utilization of the material.

## 1. Introduction

There are two primary methods of strengthening reinforced concrete (RC) members in flexure with fiber reinforced polymer (FRP) materials: laminates externally bonded (EB) to the tensile surface of the concrete and the near surface mounted (NSM) method in which narrow, typically carbon FRP (CFRP) strips or bars are embedded into slots made in the concrete cover. Although, EB FRP strengthening has been widely used, it has poor strengthening efficiency (in terms of utilizing the FRP material strength) due primarily to premature debonding from the concrete surface. Pretensioning EB FRP increases the efficiency of the system by increasing the FRP strain that is attained prior to debonding, but does not mitigate debonding failure. The NSM method was proposed to mitigate FRP debonding and allow the full utilization of FRP material strength. Strengthening with FRP materials that are pretensioned prior to bonding is an efficient technique to increase the load bearing capacity of the RC members and to improve performance at the serviceability limit state (SLS), specifically increasing the cracking moment and reducing deflections under service loads. Pretensioning the FRP effectively reduces existing crack widths, delays their further development, decreases existing deflections, and relieves stress in the internal reinforcement. The influence of many parameters on the strengthening efficiency of the NSM method have been investigated, including the CFRP pretensioning strain, internal reinforcement ratio, concrete strength, and the CFRP reinforcement length and elastic modulus [1,2,3,4,5,6,7,8,9,10,11,12,13]. Review of the available literature on flexural strengthening of RC members with passive and pretensioned NSM materials concludes that the strengthening efficiency depends on a number of factors, including the internal steel reinforcement ratio, the pretensioning limit, defined as the level of CFRP pretension as a ratio of the ultimate CFRP strain, and the pretensioning method. Although the CFRP pretensioning limit affects the serviceability of FRP-strengthened structures, it has no influence on the increase in load-carrying capacity attributed to the CFRP. From a review of published research, an increase of the load bearing capacity is affected by the existing longitudinal steel reinforcement ratio [2]. The increase in the capacity of prestressed RC members ranges from 11.5% for specimens with an internal steel reinforcement ratio of *ρ*_s_ = 1.75% [4] to 152% for specimens with a very low internal reinforcement ratio of *ρ*_s_ = 0.35% [5]. An increase in the steel reinforcement ratio results in a decrease in the degree of strengthening that can be achieved. Another investigated parameter was the pretensioning level of the CFRP laminates [2,3,5,6,7,8,9,10,11]. The maximum pretensioning level studied was 60% of the tensile strength of the CFRP [2,5]. However such a high pretensioning level did not lead to an increase in load capacity in comparison to specimens pretensioned to 40% of the CFRP tensile strength. Rather, the higher pretension led to a decrease in the ultimate load capacity due to premature rupture of the CFRP strips. The most effective pretensioning level of NSM CFRP strips was determined to fall in the range of 20% and 30% of the tensile strength [5]. The greatest benefit of the NSM pretensioning technique is in preventing premature debonding of the FRP from the concrete surface [6], which is the dominant limit state for EB FRP laminates.

Although preloading is one of the most important parameters to be taken into account in strengthening existing RC structures, this issue has been investigated very rarely. To the authors’ knowledge, no research results have been published on the effects of initially preloaded RC members strengthened with pretensioned NSM FRP strips.

In the experimental tests presented in this paper a novel system for pretensioning narrow NSM CFRP strips embedded in the concrete cover was used [14]. The aim of the experimental program was to evaluate the strengthening efficiency of the pretensioned NSM method. The study focused on the influence of the internal steel reinforcement ratio and the number of pretensioned strips used on the strengthening effectiveness. The presented research also presents an analysis of the effect of preloading on strengthening efficiency.

## 2. Experimental Program

**Figure 1:**
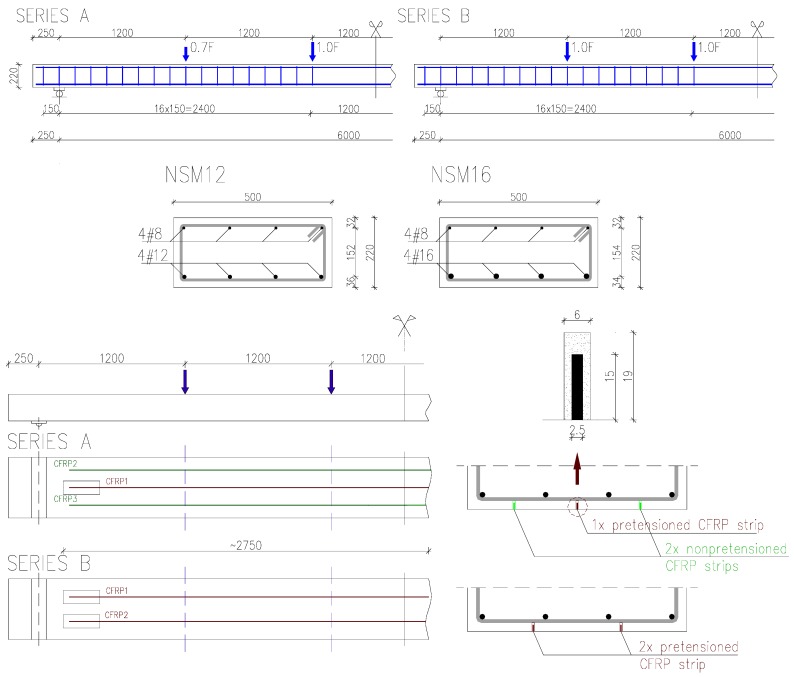
Static schemes, specimens’ cross-sections, and strengthening configurations.

The experimental program comprised six 500 × 220 mm^2^ rectangular RC beams having a simply-supported span of 6000 mm. Shear reinforcement consisted of 8-mm-diameter steel stirrups with 150-mm spacing. The beams were divided into two series: A and B, depending on the number of NSM CFRP strips. Single-span simply-supported beams were tested in static six point loading with load points spaced at 1200 mm across the span, as shown in Figure 1. Loading was introduced using two 100 kN hydraulic jacks and transferred to the beams through a steel spreader beam.

Each specimen was tested to failure. Specimens were labelled as follows: NSM12 and NSM16—beams reinforced with 12 and 16 mm diameter longitudinal steel bars, respectively; A or B—Beam of series A or series B; L—External preloading of the beam prior to strengthening.

Series A consisted of two beams (NSM12A and NSM16A) strengthened with a combination of one pretensioned and two passive CFRP strips. Series B included four specimens (NSM12B, NSM16B, NSM12B-L and NSM16B-L), each strengthened with two pretensioned CFRP strips. The internal tensile steel reinforcement consisted of four steel bars with a nominal diameter of 12 mm (*ρ*_s_ = 0.49%) in beams labelled NSM12, and four bars with a diameter of 16 mm (*ρ*_s_ = 0.87%) in beams labelled NSM16.

Beams were strengthened with rectangular CFRP strips 2.5 mm wide and 15 mm high, resulting in a composite reinforcement ratio equal to *ρ*_f_ = 0.10% and *ρ*_f_ = 0.07%, in series A and series B, respectively. The pretensioning force was applied to the CFRP strip with strain control to ε_fp_ = 0.006, corresponding to 33% of the ultimate tensile strength of the CFRP (0.33*f*_fu_).

The beams were strengthened under two preloading levels. The first was the self-weight of the beam only, corresponding to the stresses in the longitudinal reinforcing steel of 25% and 14% of the yield strength in the unstrengthened members (*M*_u0_) for the beams NSM12 and NSM16, respectively. The maximum preloading level, corresponding to 60% of the steel yield in a unstrengthened beam (*M*_u0_), was applied using the self-weight and external preloading using the loading pattern shown in Figure 1. The resulting moment associated with preloading, *M*_p_, is given in Table 1.

The beams were fabricated using commercially-supplied class C50/60 concrete. The average compressive strength of concrete and the modulus of elasticity (*E*_cm_) were defined at the day of testing using uniaxial compression tests on 150 × 300 mm^2^ cylinders (*f*_ck_) and 150 mm cube samples (*f*_c,cube_) [15], while the tensile strength (*f*_ct,sp_) was determined based on the splitting test for 150 mm cube samples [16]. All material test specimens were cured under the same conditions as the tested beams. Concrete properties are reported in Table 2. The tensile characteristics of longitudinal steel bars are presented in Table 2 [17]. The average experimentally-determined strength characteristics of CFRP strips [18]: tensile strength (*f*_fu_), elastic modulus (*E*_f_), and ultimate strain (ε_fu_) are also reported in Table 2. Strength properties according to the manufacturer are also shown Table 2. 

**Figure 2:**
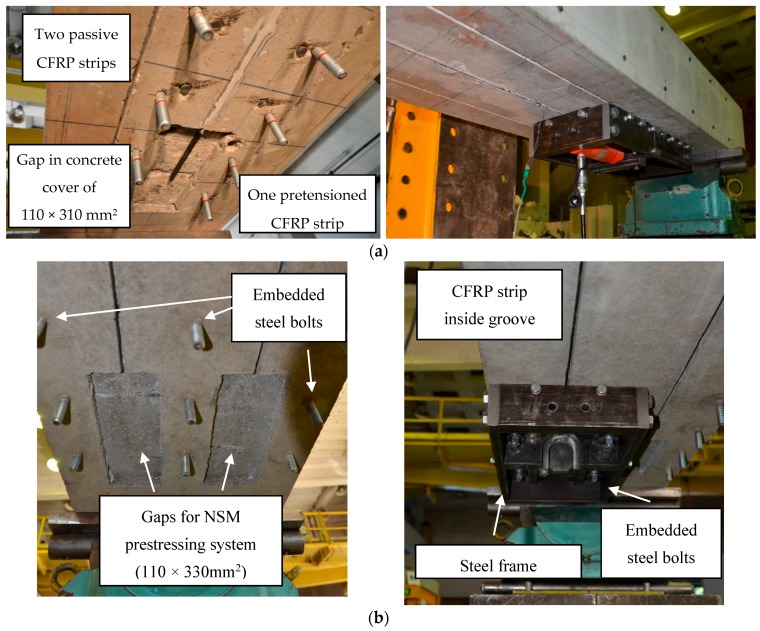
Preparation of strengthening: (**a**) beams series A; and (**b**) beams series B.

The beams were strengthened with CFRP laminates bonded into slots with a commercially-available two-component (3:1) epoxy adhesive intended for the purpose. In order to affect preload, the strengthening process was undertaken in the test frame with the beam in its correct position; that is, the NSM strips were installed overhead. The beams were strengthened under loading (consisting of the dead load and/or external loading as indicated in Table 1). Prior to strengthening, two (series B) or three (series A) slots were cut using a diamond saw blade. Each slot was 6 mm wide and 19 mm deep. To accommodate the pretensioning hardware, the slots prepared for the pretensioned laminates terminated with a 110 mm × 300 mm rectangular region having a depth of 19 mm (Figure 2).

**Figure 3:**
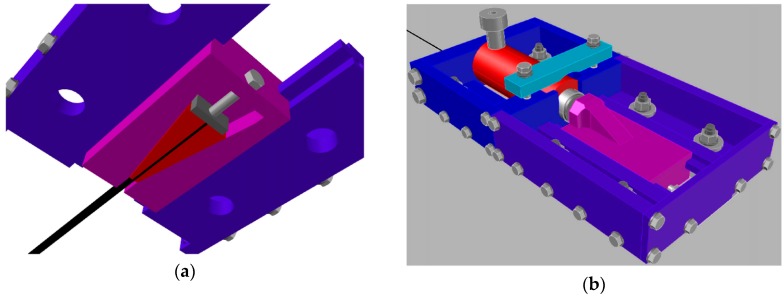
NSM pretensioning system for CFRP strips: (**a**) top view (against the beam soffit); and (**b**) bottom view.

After preparing the slots, steel bolts were installed on the bottom of the beam for the NSM pretensioning system. The CFRP strips were mounted in the pretensioning system and hydraulic jacks were mounted at both pretensioning frames at the strip’s ends (Figure 3). The initial pretensioning force was strain-controlled, intended to be equal to ε_fp_ = 0.006, corresponding to 33% of the CFRP tensile strength.

Beams of series A were strengthened with one pretensioned CFRP strip. The middle slot was filled with adhesive and the CFRP strip was embedded into the slot. A 300 mm length at each of the slots was cured at 90 °C for 45 min. The remainder of the strip was not subject to accelerated cure. As the anchoring adhesive cured, the pretensioning force was reduced by approximately 50%. After pretensioning of the middle CFRP strip (Figure 1), it was anchored to clamps mounted in the gaps made in the concrete cover for an additional 12 h prior to complete release. Releasing the pretension force in this incremental manner was intended to ensure the proper transfer of the tensile force from the CFRP strip to concrete. Two non-pretensioned CFRP strips were bonded into the remaining slots. The combination of active and passive strengthening was used due to the difficulty of using the NSM pretensioning system for all three strips due to the lack of space on the bottom surface of the specimens. NSM spacing of 160 mm is required to effectively use the pretensioning system for adjacent strips.

In the beams of series B, strengthened with two pretensioned CFRP strips, the strips were installed and pretensioned one after another. The pretensioning process proceeded in the same manner as described for series A.

**Figure 4:**
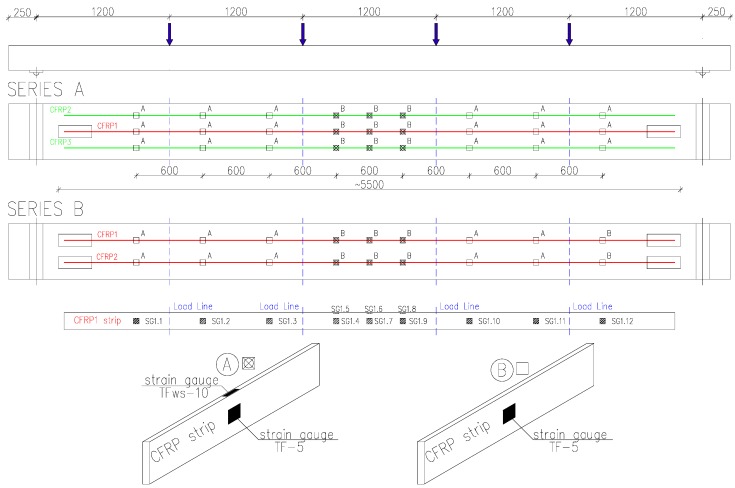
Strain gauges on CFRP strips (**A**—Bottom and lateral side; **B**—Lateral side).

**Figure 5:**
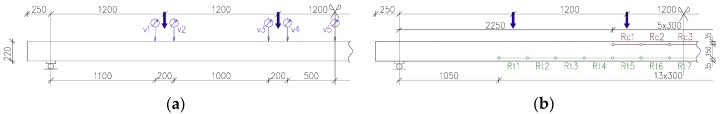
Location of LVDT gauges: (**a**) vertical displacement; and (**b)** horizontal concrete strain.

To monitor the strain distribution along the full length of the CFRP strip, twelve strain gauges were mounted on each strip at the locations shown in Figure 4. Vertical displacements of the beams were recorded with nine 50 mm stroke linear variable differential transformers (LVDTs) (Figure 5a). The concrete strains were measured with horizontal LVDTs arranged over 300 mm gauge lengths (13–20 mm stroke gauges in the tensile zone and 5–10 mm stroke gauges in the compressive zone), as shown in Figure 5b.

## 3. Test Results

### 3.1. Failure Modes

**Figure 6:**
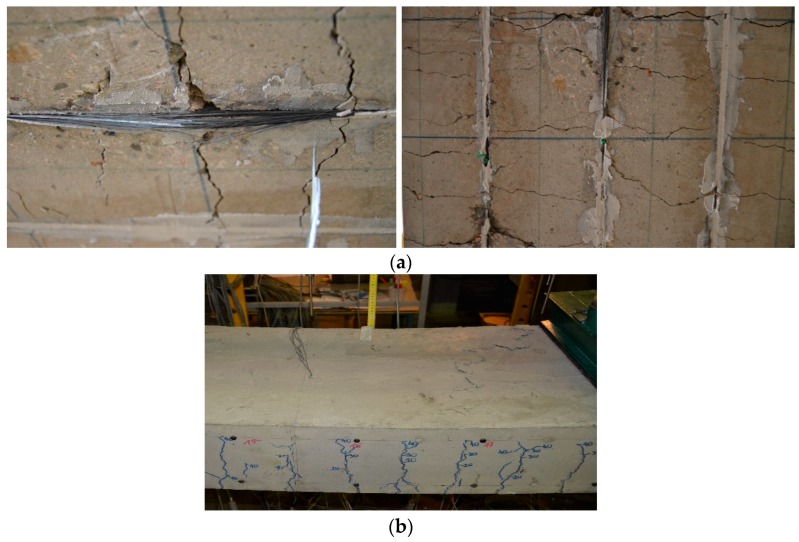
Failure of the specimens in series A: (**a**) bottom view of NSM12A with rupture of CFRP strips; and (**b**) the top view of NSM16A with concrete crushing.

All beams failed in flexure due to rupture of both pretensioned and passive NSM CFRP strips preceeded by sounds of gradually-breaking carbon fibers (Figure 6a). This failure mode confirmed the full utilization of the CFRP tensile strength, in both pretensioned and passive strips. The single pretensioned strip in series A ruptured first in both members (NSM12A and NSM16A). Further load increase led to the rupture of the remaining two passive strips. Concrete crushing in the compression zone occurred in beam NSM16A (having a higher reinforcement ratio), as shown in Figure 6b.

### 3.2. Cracking Pattern

**Figure 7:**
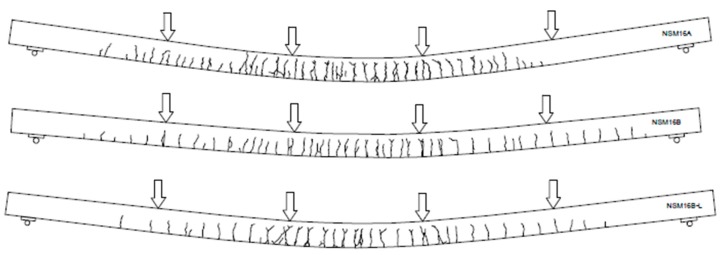
Cracking pattern of specimens with the higher reinforcement ratio (NSM16).

**Figure 8:**
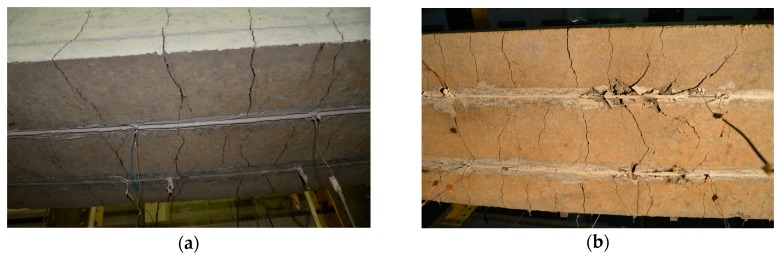
Cracking pattern on the bottom surface of beams of series B: (**a**) NSM12B; and (**b**) NSM12B-L.

Concrete cracking in all tested beams was monitored during the full range of loading. Irrespective of preloading level, crack patterns were similar in all beams, as seen in Figure 7. Differences in the cracking pattern after failure were caused by the different composite reinforcement ratio of each series and by the number of pretensioned CFRP strips. The beams strengthened with two pretensioned laminates exhibited fewer cracks than the beams strengthened with one pretensioned and two non-pretensioned CFRP strips. No longitudinal cracks in the concrete along the slot, at the level of the bottom steel reinforcement, or at the epoxy-concrete interface were observed, confirming the very good bond behavior of the NSM CFRP strips to concrete through the full range of loading. The “fishbone” cracking pattern on the bottom surface of the beams, typical for NSM strengthening [19], was observed in all beams (Figure 8a,b). This cracking pattern confirms progressive tensile stress transfer from the pretensioned strips to the concrete substrate.

## 4. Discussion of the Test Results

Analysis of strengthening efficiency for all beams was performed for three loading levels corresponding to concrete cracking, steel yielding, and ultimate load. Values of the bending moment of the unstrengthened the strengthened beams, corresponding to these levels, are summarized in Table 3. Bending moments for the unstrengthened beams were calculated using a nonlinear analytical model, which considers normal stresses in the cross-section, experimental stress-strain characteristics of concrete and steel using the plane sections principle, and accounting for tension stiffening in the full range of loading. Details of the model were described and applied for similar RC beams in [19].

The strengthening ratio was determined as a percentage of the difference between the ultimate bending moment of the strengthened (*M*_u_) and unstrengthened (*M*_u0_) beam to the ultimate bending moment of the unstrengthened member (*M*_u0_) as follows:

Δ*M*_u_ = 100 × (*M*_u_ − *M*_u0_)/*M*_u0_ (1)

Test results confirmed a common opinion about dependence of the strengthening efficiency on the internal steel reinforcement ratio. Beams with a lower steel reinforcement ratio (NSM12) demonstrated the greatest increase of load bearing capacity: ranging from 97% and 135%, while the beams with the higher reinforcement ratio (NSM16) exhibited only about one-half improvement: ranging from 56% to 70.8%. The research revealed a very interesting result with respect to the preloading effect, which had an insignificant influence on the load bearing capacity. This conclusion is very promising for practical applications of NSM strengthening even for structures having relatively high dead load to residual capacity ratios.

The increase in the cracking moment was more affected by the number of pretensioned CFRP strips than by a total number of the NSM strips. This was confirmed by an increase in the cracking moment of 69.2% for beam NSM12A (strengthened with one pretensioned strip and two non-pretensioned ones) and 100% for the beam NSM12B (strengthened with two pretensioned strips). However, for the beams with the higher internal reinforcement ratio this increase was lower and equal to 82% and 93.3% for the beam NSM16A and NSM16B (Table 3).

The steel yielding moment was sensitive to the total number of strips only in the beams with a lower reinforcement ratio reaching increases of the steel yielding moment of 50.5% and 24%, respectively, for the beams strengthened with three (NSM12A) and two CFRP strips (NSM12B) relative to the analytically-determined value of yield capacity. No influence of the number of strips on the steel yielding bending moment was observed in beams NSM16A and NSM16B, which confirms the influence of the internal steel reinforcement ratio on the steel yielding moment (Table 3).

In the specimens of series A, the CFRP strains after pretensioning stabilized at values of 0.0054 and 0.0061, respectively, for beams NSM12A and NSM16A (Table 4). While in the specimens strengthened with two pretensioned CFRP strips (series B), the CFRP pretensioning strains stabilized when they reached values of 0.0046 and 0.0054, respectively, for beams with lower (NSM12B) and higher (NSM16B) internal reinforcement ratios.

The ultimate strains in the CFRP strips in the beams of series A reached values of 0.0186 and 0.0171 in the pretensioned CFRP strips and between 0.0149 and 0.0179 in the non-pretensioned CFRP strips. Similarly, in the specimens of series B the maximum strains in the tests ranged between 0.0147 and 0.0183.

**Figure 9:**
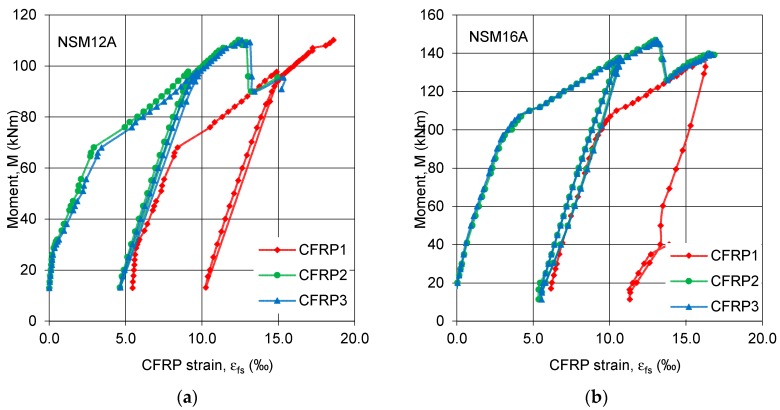
Bending moment vs. CFRP strain curves in beams of series A: (**a**) NSM12A; and (**b**) NSM16A.

The CFRP strips in the beams of series A deformed uniformly across the section, as shown by strain measurements on all three CFRP strips. Irrespective of the internal steel reinforcement ratio (NSM12, NSM16) or type of the CFRP strip (pretensioned (CFRP1), non-pretensioned (CFRP2, CFRP3)), the slopes of the CFRP strain curves as functions of the bending moment were very similar (Figure 9). The offset of the curve for CFRP1 results from the pretensioning of this strip to 0.0054 and 0.0061 in beams NSM12A and NSM16A, respectively. From the comparison of strain curves of pretensioned (CFRP1) and non-pretensioned (CFRP2, CFRP3) strips the moment when the pretensioned strip ruptured, resulting in stress redistribution to the remaining two strips took over is evident (see the curves of CFRP2 and CFRP3 with an abrupt drop of the bending moment at strains equal to 0.0149 and 0.0164, respectively, in beam NSM12A, and at 0.0179 and 0.0177 in beam NSM16A).

**Figure 10:**
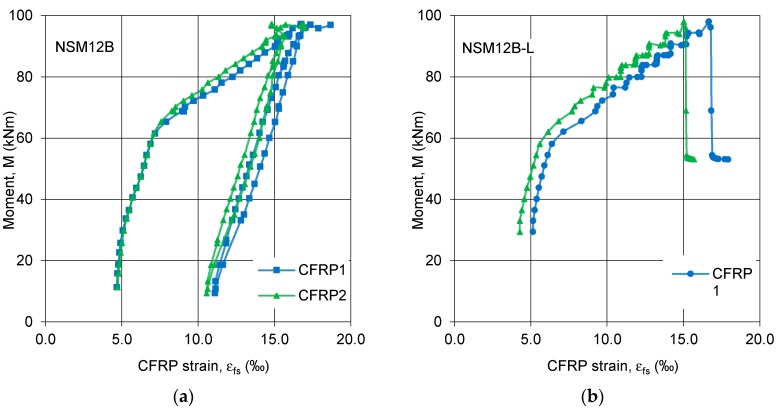
Bending moment vs. CFRP strain curves of series B: (**a**) NSM12B; and (**b**) NSM12B-L.

**Figure 11:**
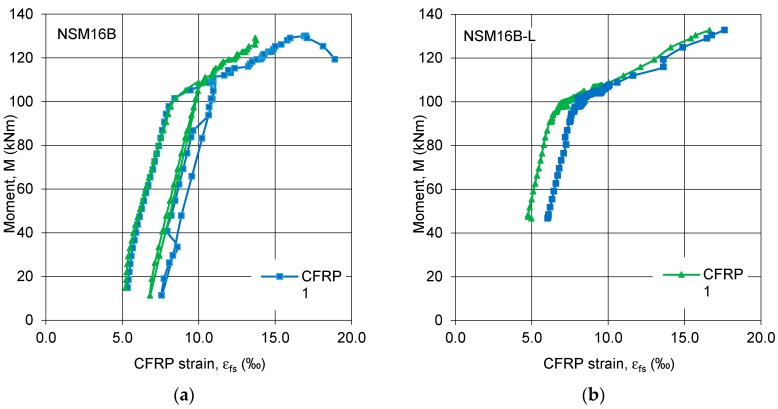
Bending moment vs. CFRP strain curves of series B: (**a**) NSM16B; and (**b**) NSM16B-L.

The strain curves of the pretensioned CFRP strips in the beams of series B, strengthened under only self-weight (NSM12B) demonstrated uniform deformation of both strips (Figure 10a), while the corresponding strain curves in the beam preloaded before strengthening (NSM12B-L) shows a difference in the CFRP strains (Figure 10b). The vertical translation of the curves in Figure 10 and Figure 11 represents the bending moment caused by preloading the specimens. The difference in strains seen in Figure 10b was caused by the step-wise bonding the pretension strips. After installation of the first strip, the second was pretensioned and bonded resulting in an additional prestress applied to the beam and a resulting decrease in the pretension of the first strip. This difference in the beam NSM16B-L is reduced when the internal steel starts to yield (Figure 11b). However, in the beam NSM12B-L the difference in strain curves between strips remains for the full range of loading. The difference between NSM12B-L and NSM16B-L is due to the relative contribution of the CFRP strips in resisting the tensile forces: the CFRP contribution in beam NSM12B-L, having a lower internal reinforcement ratio, is greater than that of beam NSM16B-L.

Pretensioning the strips resulted in a camber (negative vertical displacement) occurring during the pretensioning process. Values of camber resulting from pretensioning (*v*_p_), bending moment *M*_(L/200)_, corresponding to the allowable deflection based on the serviceability limit state (*v*_a_ = 30 mm), and the deflection corresponding to the ultimate bending moment, *v*_M(max)_, are summarized in Table 4. The beams strengthened with one pretensioned strip (series A) differed in their internal reinforcement ratio, but exhibited a camber equal to 1.9 and 1.7 mm, respectively, for beams NSM12A and NSM16B. As expected, beams strengthened with two pretensioned strips exhibited greater camber: 6.4 mm for beam NSM12B and 3.6 mm for beam NSM16B. The beams preloaded before strengthening were cracked and, therefore, exhibited a reduced stiffness, which led to greater camber following pretensioning: 7.7 and 5.2 mm, respectively, for beams NSM12B-L and NSM16B-L (Table 4).

**Figure 12:**
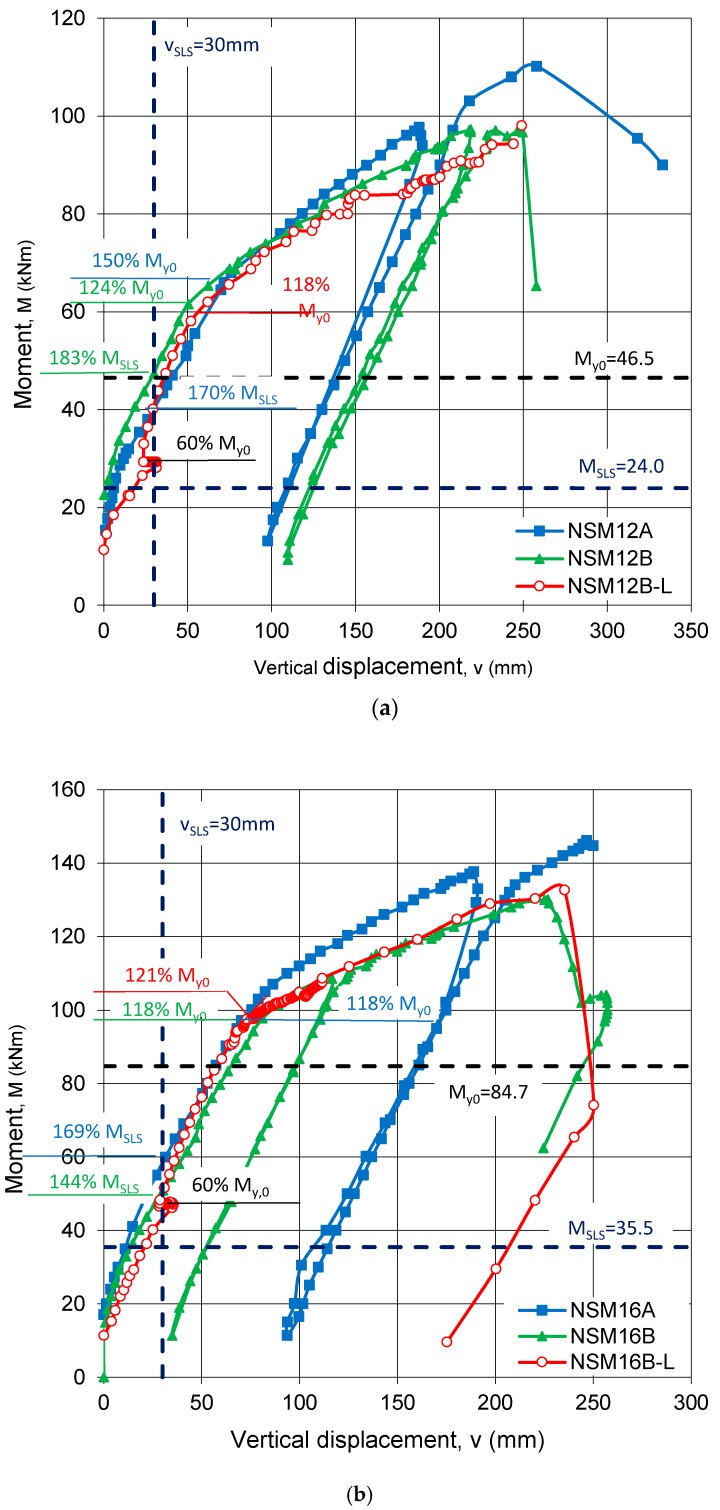
Bending moment vs. vertical displacement curves: (**a**) Series A; and (**b**) Series B.

A comparison of vertical displacements for the beams with lower and higher reinforcement ratios are shown in Figure 12a,b, respectively. The vertical translation of the curves in Figure 12 represents the bending moment caused by preloading the specimens. The curves of the beams pretensioned under only self-weight are very similar before the steel yields, however, they diverge after steel yielding. The similar behavior before yield illustrates that the presence of NSM CFRP, regardless of amount, has little effect on the uncracked and partially-cracked (i.e., pre steel yield) stiffness of the cross-sections. Following the yield of the internal reinforcement, the CFRP provides all tensile stiffness and, therefore, beam stiffness is expected to be greater for the beam strengthened with three (NSM12A), rather than two, CFRP strips (NSM12B). The ultimate capacity is similarly affected: beam NSM12B failed at a lower ultimate load than beam NSM12A. Similar observations are made from the load-displacement curves of the displacement for the beams reinforced with a higher reinforcement ratio (NSM16). The greater external preloading did not influence the flexural behavior of the tested specimens (NSM12B-L and NSM16B-L).

Following a decrease in the existing deflections due to prestressing in the specimens NSM12B-L and NSM16B-L (v_p_ in Table 4), the slope of the bending moment vs. deflection curves is the same as those of the beams strengthened under only self-weight. In Figure 12, a dashed vertical line shows the allowable deflection at the service limits state (L/200 = 30 mm). The increase in the bending moment corresponding to the allowable deflection ranged from 70% and 83% for beams NSM12A and NSM12B, and from 44% and 69% for beams NSM16B and NSM16A. This confirms a beneficial influence of prestressing on the overall flexural behavior and stiffness of the beams initially preloaded under only self-weight. The displacement vs. bending moment curves for both NSM12B-L and NSM16B-L beams confirm the beneficial effect of prestressing, which is a very promising conclusion for practical application of NSM strengthening even for structures having relatively high dead load to residual capacity ratios.

## 5. Conclusions

This study provided several important conclusions concerning the factors influencing the efficiency of pretensioned CFRP strips for strengthening reinforced concrete members:

- The high efficiency of the pretensioning technique for flexural strengthening with NSM CFRP was confirmed by high strengthening ratios, ranging from 56% to 135%. The strengthening ratio that may be achieved is shown to be inversely proportional to the existing internal steel reinforcement ratio.
- A different preloading history for beams NSM12B-L and NSM16B-L did not affect the flexural behavior of these specimens during subsequent loading.
- Pretensioning of the CFRP reinforcement enhances the beam performance at the serviceability limit state. This was demonstrated by an increase of bending moment corresponding to the allowable deflection of 70%. This also applies to members having large preload prior to strengthening. Moreover, it is the overall composite reinforcement ratio, rather than number of pretensioned strips, that effects the final increase in load bearing capacity.
- The results of the tests indicated high efficiency of the novel pretensioning system invented by the authors, which makes it possible to apply NSM CFRP without any anchorage systems.

## 6. Patents

The research disseminate the Patent P.407898, 14 April 2014.

## Figures and Tables

**Table 1:** Summary of tested beams, investigated parameters, and strengthening configuration.

Specimen	Tensile Steel Reinforcement	Number of CFRP Strips	Number of Pretensioned CFRP Strips	*M*_u0_^1^ [kNm]	*M*_p_^2^ [kNm]	*M*_p_/*M*_u0_^3^ [%]
NSM12A	4ϕ12	3	1	46.5	13.5	25
NSM16A	4ϕ16	3	1	84.7	15.0	14
NSM12B	4ϕ12	2	2	46.5	13.5	25
NSM16B	4ϕ16	2	2	84.7	15.0	14
NSM12B-L	4ϕ12	2	2	46.5	27.9	60
NSM16B-L	4ϕ16	2	2	84.7	50.9	60

^1^*M*_u0_—Analytically-determined moment bearing capacity of the unstrengthened specimen, ^2^*M*_p_—Preloading bending moment, ^3^*M*_p_/*M*_u0_—Preloading ratio.

**Table 2:** Strength characteristics of used materials.

Material	Property	Unit	Series A				Series B			
			NSM12		NSM16		NSM12		NSM16	
			8	12	8	16	8	12	8	16
Steel	*E*_s_	[GPa]	186.1	191.3	196.5	198.0	205.5	214.0	205.5	204.9
	*f*_y_	[MPa]	416.2	539.6	555.8	595.0	554.9	563.4	554.9	578.3
	*f*_u_	[MPa]	734.1	627.5	646.0	672.0	608.9	651.7	608.9	693.8
Concrete	*f*_ck_	[MPa]	46.0		53.9		51.0		52.0	
	*f*_c,cube_	[MPa]	44.9		59.5		60.0		60.1	
	*f*_ct,sp_	[MPa]	3.95		4.30		4.5		4.1	
	*E*_cm_	[GPa]	25.3		24.0		25.8		24.3	
CFRP	*E*_f_	[GPa]	170.4 (manufacturer reports 160)							
	*f*_fu_	[MPa]	2551 (manufacturer reports 2800)							
	ε*_fu_*	[–]	0.0136 (manufacturer reports 0.017)							

**Table 3:** Summary of test results.

Specimen	Failure Mode	*M*_p_/*M*_u0_ [%]	*M*_cr0_ [kNm]	*M*_cr_ [kNm]	Δ*M*_cr_ [%]	*M*_y0_ [kNm]	*M*_y_ [kNm]	Δ*M*_y_ [%]	*M*_u0_ [kNm]	*M*_u_ [kNm]	Δ*M*_u_ [%]
NSM12A	R ^1^	25	13.0	22.0	69.2	46.5	70.0	50.5	46.7	110.2	135.0
NSM12B	R	25	13.0	26.0	100.0	49.5	61.5	24.0	49.7	97.0	95.2
NSM12B-L	R	60	13.0	-	-	49.5	58.5	18.0	49.7	98.0	97.0
NSM16A	R + CC ^2^	14	15.0	27.3	82.0	85.2	100.0	18.0	86.0	146.9	70.8
NSM16B	R	14	15.0	29.0	93.3	83.0	98.0	18.1	83.3	130.0	56.1
NSM16B-L	R	60	15.0	-	-	83.0	100.1	21.0	83.3	133.0	59.7

^1^ R—Rupture of CFRP, ^2^ CC—Concrete crushing.

**Table 4:** Summary of CFRP strain and deflections after pretensioning.

Specimen	Preloading	CFRP Strain after Pretensioning				*v*_p_ [mm]	*M*_(L/200)_ [kNm]	Δ*M*_(L/200)_ [%]	*v*_M(max)_ [mm]
		ε_fp_ [‰]		σ_fp_ [MPa]					
NSM12A	0.25*M*_u0_	5.4		864		−1.9	41.0	70.0	258
NSM16A	0.14*M*_u0_	6.1		976		−1.7	51.0	44.0	245
NSM12B	0.25*M*_u0_	4.6	4.7	736	752	−6.4	44.0	83.0	256
NSM12B-L	0.60*M*_u0_	5.1	4.3	816	688	−7.7	26.0	-	250
NSM16B	0.14*M*_u0_	5.4	5.4	864	704	−3.6	60.0	69.0	230
NSM16B-L	0.60*M*_u0_	5.2	6.1	826	973	−5.2	35.0	-	236

## References

[1] Zhang S.S., Yu T., Chen G.M. (2017). Reinforced concrete beams strengthened in flexure with near-surface mounted (NSM) CFRP strips: Current status and research needs. Compos. Part B.

[2] Badawi M., Soudki K. (2009). Flexural strengthening of RC beams with prestressed NSM CFRP rods—Experimental and analytical investigation. Constr. Build. Mater..

[3] Choi H.T. (2008). Flexural Behaviour of Partially Bonded CFRP Strengthened Concrete T-Beams. Ph.D. Thesis.

[4] El-Hacha R., Rojob H. FRP prestressing systems for flexural strengthening of structural elements—A review. Proceedings of the 8th International Conference on FRP Composites in Civil Engineering.

[5] Gaafar M.A., El-Hacha R. Strengthening reinforced concrete beams with prestressed near surface mounted FRP strips. Proceedings of the 4th International Conference on FRP Composites in Civil Engineering (CICE2008).

[6] Gaafar M.A., El-Hacha R. (2011). Flexural strengthening of reinforced concrete beams using prestressed, near surface mounted CFRP bars. PCI J..

[7] Hajihashemi A., Mostofinejad D., Azhari M. (2011). Investigation of RC beams strengthened with prestressed NSM CFRP laminates. J. Compos. Constr..

[8] Hosseini M.M., Dias S.J., Barros J.A. (2014). Effectiveness of prestressed NSM CFRP laminates for the flexural strengthening of RC slabs. Compos. Struct..

[9] Hosseini M.M., Dias S.J., Barros J.A. (2016). Flexural strengthening of reinforced low strength concrete slabs using prestressed NSM CFRP laminates. Compos. Part B.

[10] Nordin H., Taljsten B. (2006). Concrete Beams Strengthened with Prestressed Near Surface Mounted CFRP. J. Compos. Constr..

[11] Rezazadeh M., Costa I., Barros J. (2014). Influence of prestress level on NSM CFRP laminates for the flexural strengthening of RC beams. Compos. Struct..

[12] Lee H.Y., Jung W.T., Chung W. (2017). Flexural strengthening of reinforced concrete beams with pre-stressed near surface mounted CFRP systems. Compos. Struct..

[13] Al-Saadia N.T.K., Mohammeda A., Al-Mahaidia R. (2018). Bond performance of NSM CFRP strips embedded in concrete using direct pull-out testing with cementitious adhesive made with graphene oxide. Constr. Build. Mater..

[14] Kotynia R., Lasek K. (2014). Anchoring and pretensioning system for the plates in particular near surface mounded composite strips. PŁ Patent.

[15] (2011). Testing Hardened Concrete. Compressive Strength of Test Specimens.

[16] (2011). Testing Hardened Concrete. Tensile Splitting Strength of Test Specimens.

[17] (2014). Steel for the Reinforcement and Prestressing of Concrete—Test Methods—Part 1: Reinforcing Bars, Wire Rod and Wire.

[18] (2004). Guide Test Methods for Fiber-Reinforced Polymers (FRPs) for Reinforcing and Strengthening Concrete Structures.

[19] Kotynia R., Lasek K., Staskiewicz M. (2013). Flexural behavior of preloaded (RC) slabs strengthened with prestressed CFRP laminates. J. Compos. Constr..

